# Effects of Sr/F-Bioactive Glass Nanoparticles and Calcium Phosphate on Monomer Conversion, Biaxial Flexural Strength, Surface Microhardness, Mass/Volume Changes, and Color Stability of Dual-Cured Dental Composites for Core Build-Up Materials

**DOI:** 10.3390/nano12111897

**Published:** 2022-06-01

**Authors:** Bharat Mirchandani, Chawal Padunglappisit, Arnit Toneluck, Parichart Naruphontjirakul, Piyaphong Panpisut

**Affiliations:** 1 Faculty of Dentistry, Thammasat University, Pathum Thani 12120, Thailand; drbharat@tu.ac.th (B.M.); pchawal@tu.ac.th (C.P.); rnit_pook@hotmail.com (A.T.); 2 Biological Engineering Program, Faculty of Engineering, King Mongkut’s University of Technology Thonburi, Bangkok 10140, Thailand; parichart.nar@kmutt.ac.th; 3 Thammasat University Research Unit in Dental and Bone Substitute Biomaterials, Thammasat University, Pathum Thani 12120, Thailand

**Keywords:** core build-up, composite resins, bioactive glass, calcium phosphate, polymerization, flexural strength, mass/volume changes, color stability, surface microhardness

## Abstract

This study prepared composites for core build-up containing Sr/F bioactive glass nanoparticles (Sr/F-BGNPs) and monocalcium phosphate monohydrate (MCPM) to prevent dental caries. The effect of the additives on the physical/mechanical properties of the materials was examined. Dual-cured resin composites were prepared using dimethacrylate monomers with added Sr/F-BGNPs (5 or 10 wt%) and MCPM (3 or 6 wt%). The additives reduced the light-activated monomer conversion by ~10%, but their effect on the conversion upon self-curing was negligible. The conversions of light-curing or self-curing polymerization of the experimental materials were greater than that of the commercial material. The additives reduced biaxial flexural strength (191 to 155 MPa), modulus (4.4 to 3.3), and surface microhardness (53 to 45 VHN). These values were comparable to that of the commercial material or within the acceptable range of the standard. The changes in the experimental composites’ mass and volume (~1%) were similar to that of the commercial comparison. The color change of the commercial material (1.0) was lower than that of the experimental composites (1.5–5.8). The addition of Sr/F-BGNPs and MCPM negatively affected the physical/mechanical properties of the composites, but the results were satisfactory except for color stability.

## 1. Introduction

Dental composites are usually placed as dentine substitute material (core build-up restoration) for severely damaged teeth that require complex direct or indirect restorations [1]. The use of composites that can be polymerized via both light-activated and chemical-activated polymerization (dual-cured) is beneficial to ensure sufficient polymerization of the materials in a deep cavity [2,3]. The placement of definitive restorations for teeth temporized with core build-up materials may be delayed in some circumstances, such as ongoing orthodontic treatments or patients with limited budget/cooperation. The main concern of an extensive cavity restored with resin composite is the risk of bacterial microleakage and demineralization at tooth-composite margins [4,5]. This may subsequently cause secondary caries and severe infections that require complicated treatments or extraction. The main limitations of the commonly used dual-cured resin composites for core build-up included the lack of ion-releasing and antibacterial action to inhibit biofilm formation and tooth-demineralization [6].

Several types of calcium phosphates (CaP) were used as the reactive fillers to encourage ion release for promoting anti-caries actions. It was demonstrated that incorporating CaP with a low Ca/P ratio, such as monocalcium phosphate monohydrate (MCPM, promoted ion release and mineralizing actions for resin composites [7,8]. However, the addition of MCPM may significantly decrease the physical/mechanical properties of the materials. This may consequently compromise the strength and longevity of the restorations. The previous study reported that the incorporation of MCPM reduced the biaxial flexural strength of the experimental resin composites by approximately 20% [9,10].

Bioactive glass nanoparticles represent an alternative ion-releasing filler [11]. Several studies have incorporated sol-gel-derived bioactive glass nanoparticles into resin composites to enable ion releasing or antibacterial actions for the materials [12,13,14,15]. Additionally, it was reported that the addition of bioactive glass nanoparticles exhibited no significant effects on the mechanical strength of the composites [16,17,18]. Moreover, bioactive glass produced in a spherical shape exhibited a more remarkable mineralizing ability than that of the glass produced as irregular/granular particles [19]. It was demonstrated that the use of nanosized glass with a spherical shape (diameter ~ 200 nm) provided color structural effects to enhance the optical properties of the materials [20]. The bioactive glass contained multiple ions in its glass network. The addition of bioactive glass containing fluoride may promote the precipitation of acid-resistant fluorohydroxyapatite for the tooth structure [21]. The combination of strontium with fluoride ions also demonstrated synergistic effects on anti-caries actions [22,23]. Furthermore, bioactive glass containing Sr exhibited greater antibacterial actions than non-Sr-doped bioactive glass [24]. It was also demonstrated that Sr^2+^ might increase nucleation clusters essential for apatite precipitation [25,26].

It was expected that a combination of MCPM and bioactive glass nanoparticles in composites might allow ion release without detrimental effects on the strength of composites [9,10]. However, the mismatch of refractive indexes between reactive fillers with resin matrix may affect the polymerization of composites due to increased light-scattering [8]. The decrease in monomer conversion could affect polymer rigidity and promote water sorption leading to polymer expansion [27]. This may subsequently lead to the reduction of strength [28], poor color stability [29], hydrolytic degradation [30], and monomer elution [31]. Furthermore, the reactive fillers were not silanized, which may affect the stress transfer within the resin matrix and increase the risk of filler degradation, thus decreasing the mechanical strength of the materials [32]. The main concern for resin-based materials is the excessive shrinkage stress generated during polymerization. Additionally, the continuation of polymerization of dual-cured resin composites by chemical-activated polymerization resulted in post-gel shrinkage in the materials [31,33]. It was reported that hygroscopic expansion due to water sorption could compensate for the polymerization shrinkage and reduce the shrinkage stress of the composites [34,35]. The addition of hydrophilic fillers may enhance water sorption to expand the polymer network. However, excessive volume expansion may increase the risk of hydrolytic degradation [36], tooth expansion, or cracks [37,38].

The objective of this study was to prepare dual-cured resin composites for core build-up restoration containing MCPM and Sr/F-bioactive glass nanoparticles (Sr/F-BGNPs), followed by the assessment of their physical/mechanical properties. The effects of the additives on the degree of monomer conversion upon light/self-curing mechanisms, biaxial flexural strength, Vickers surface microhardness, mass/volume changes, and color stability were determined. The effect of raising the concentration of MCPM and Sr/F-bioactive on the tested physical/mechanical properties was also examined. The research hypothesis was that the addition of MCPM and Sr/F-BGNPs should not affect the physical/mechanical properties of the materials.

## 2. Materials and Methods

### 2.1. Materials Preparation

#### 2.1.1. Preparation of Sr/F-Bioactive Glass Nanoparticles (Sr/F-BGNPs)

Sr/F bioactive glass nanoparticles (Sr/F-BGNPs) were synthesized using the sol-gel process under the basic condition based on the previous studies [39,40,41]. Firstly, 0.32 M of ammonium hydroxide, 6 M of Milli Q water, 0.035 M sodium fluoride (Sigma Aldrich, St. Louis, MO, USA), and 14 M of ethanol (Sigma Aldrich, St. Louis, MO, USA) were mixed and stirred at 500 rpm for 30 min in a 500 mL of Erlenmeyer Flask. Then, the solution was gradually added with tetraethyl orthosilicate (0.28 M) (TEOS, Sigma Aldrich, St. Louis, MO, USA). Next, the mixed solution was stirred for 8 h. Particles were then collected by centrifugation. They were then incorporated with calcium nitrate tetrahydrate (0.14 M) (Sigma Aldrich, St. Louis, MO, USA) and strontium nitrate (0.42 M) (Sigma Aldrich, St. Louis, MO, USA). The synthesized particles were calcined at 680 °C for 3 h with the rate of heating at 3 °C/min to allow the incorporation of calcium (Ca), strontium (Sr), sodium (Na), and fluoride (F). Then, the particles were thoroughly cleaned with ethanol.

#### 2.1.2. Preparation of Experimental Dual-Cured Resin Composites for Core Build-Up Restoration

Dual-cured composites were prepared using the methods reported in the previous studies [9,42]. The liquid phase of composites (Table 1) consisted of urethane dimethacrylate (UDMA, Sigma Aldrich, St. Louis, MO, USA), triethyleneglycol dimethacrylate (TEGDMA, Sigma Aldrich, St. Louis, MO, USA), 2-hydroxyethyl methacrylate (HEMA, Sigma Aldrich, St. Louis, MO, USA), 10-MDP (Watson International Ltd., Shanghai, China), camphorquinone (CQ, Sigma Aldrich, St. Louis, MO, USA), benzoyl peroxide (BP, Sigma Aldrich, St. Louis, MO, USA), and N, N-dimethyl-p-toluidine (DMPT, Sigma Aldrich, St. Louis, MO, USA).

The powder phase contained microparticle borosilicate glass (diameter of 0.7 and 7 μm, Esstech, Essington, PA, USA), nanoparticle borosilicate glass (diameter of 180 nm, SCHOTT UK Ltd., Wolverhampton, UK), monocalcium phosphate monohydrate (MCPM, C/P ratio of ~0.5 [43], Himed, Bethpage, NY, USA) and F/Sr-BGNPs. Five formulations of the experimental materials with high and low levels of MCPM (3 or 6 wt%) and F/Sr-BGNPs (5 or 10 wt%) were prepared (Table 2).

A scanning electron microscope (SEM, JSM 7800F, JEOL LTd., Tokyo, Japan) was used to examine the morphology and size of the Sr/F-BGNPs and MCPM. Additionally, a dispersive X-ray spectrometer (EDX, X-Max 20, Oxford Instruments, Abingdon, UK) was employed to assess the elemental composition of the fillers with the beam voltage set at 5 kV.

The initiator and activator liquids were hand-mixed with the powder phase using a powder to liquid mass ratio of 3:1. Then, the mixed initiator and activator pastes were poured into a black opaque double-barrel syringe (medmix Switzerland AG, Haag, Switzerland). The composite pastes were injected and mixed using a dispenser and mixing tip (medmix Switzerland AG, Haag, Switzerland). The commercially available dual-cure composite resin (MultiCore ^®^ Flow, Shade Light, Ivoclar Vivadent, Schaan, Liechtenstein) was the commercial comparison (Table 3).

### 2.2. Degree of Monomer Conversion (DC)

An FTIR-ATR (Nicolet iS5, Thermo Scientific, Waltham, MA, USA) (*n* = 5) was used to determine the DC of materials. The composites were injected into the metal circlip (10 × 1 mm, Springmaster Ltd., Redditch, UK) on the ATR diamond. A transparent acetate sheet was then placed over the specimen. They were then light-cured with an LED dental curing light (irradiance ~1200 mW/cm^2^, SmartLite Focus Pen Style, DENTSPLY Sirona, York, PA, USA) for 20 s. The tip of the curing light was positioned at ~1–2 mm above the composite surface. The FTIR spectra (800–4000 cm^−1^) were then obtained from the bottom surface of the specimen using the resolution of 4 cm^–1^ and 8 scans. The temperature was controlled at 25 °C. The degree of monomer conversion ($D_{c}$, %) was obtained using Equation (1) [9].

(1) $$D_{c}=\frac{100\left(\Delta A_{0}-\Delta A_{t}\right)}{\Delta A_{0}}$$

where $\Delta A_{0}$ and $\Delta A_{t}\;$ are the absorbance height of the C–O peak (1320 cm^–1^) [44] relative to the background level at 1335 cm^–1^ before curing and after curing at time t, respectively. For the DC upon chemical activation, the specimens were left on the ATR diamond without light-curing to allow the chemical-activated polymerization for 10 min. The FTIR spectra were recorded for up to 30 min. The final conversion at a late time was calculated using a linear extrapolation of DC versus the inverse time to zero [42].

### 2.3. Biaxial Flexural Strenght (BFS) and Biaxial Flexural Modulus (BFM)

The composites were loaded in a metal ring (1 × 10 mm) to produce disc specimens (*n* = 5). The composites were covered by acetate sheets and glass slides. They were light-cured for 20 s on both sides. The specimens were left for 24 h at a temperature-controlled at 25 ± 1 °C before removal from the ring. Specimens with visible defects were excluded from the test. The selected specimens were immersed in 10 mL of deionized water and incubated at a temperature-controlled at 37 ± 1 °C for 24 h and 4 weeks. Prior to the test, the specimen thickness was measured in three areas using a digital vernier caliper. The specimens were then positioned in a ball-on-ring testing jig. The test was performed using a universal testing machine (AGSX, Shimadzu, Kyoto, Japan) with a 500 N load cell and a crosshead speed set at 1 mm/min. The biaxial flexural strength (BFS, Pa) and biaxial flexural modulus (BFM, Pa) was then calculated using Equation (2) and Equation (3), respectively.

(2) $$\mathrm{BFS}\;=\frac{F}{d^{2}}\left\{\left(1+\;v\right)\left[0.485\ln\left(\frac{r}{d}\right)+0.52\right]+0.48\right\}$$

(3) $$\mathrm{BFM}\;=\left(\frac{\Delta H}{{\Delta W}_{c}}\right)\times \left(\frac{\beta_{c}d^{2}}{q^{3}}\right)$$

where F is failure load (N), d represents the thickness of disc specimens (m), r is the radius of circular support of the ball-on-ring jig (m), and v is Poisson’s ratio (0.3). Furthermore, $\frac{\Delta H}{{\Delta W}_{c}}$ represents the rate of change of the load about the central deflection or gradient of force versus the displacement curve (N/m) [15]. Additionally, $\beta_{c}$ and q represents the center deflection junction (0.5024) and the ratio of the support radius to the specimen radius, respectively. After testing, the fracture area of the representative specimens was analyzed using SEM (JSM 7800F, JEOL LTd., Tokyo, Japan) and EDX (X-Max 20, Oxford Instruments, Abingdon, UK).

### 2.4. Surface Microhardness

Disc specimens (*n* = 5) were produced according to Section 2.3 (*n* = 5). They were placed in deionized water (10 mL) (*n* = 5). The specimens were incubated at 37 °C for 24 h and 4 weeks. A microhardness tester (FM-800, Future-Tech Corp, Kanagawa, Japan) was used to assess the Vickers surface microhardness of the materials [45]. The indentation load was set at 300 g with a dwelling time of 10 s. The Vickers microhardness number (VHN) was then obtained by averaging 5 different areas on the sample.

### 2.5. Mass and Volume Changes

Mass and volume of the specimens were recorded using a 4-figure balance equipped with a density kit (MS-DNY-43, METTLER TOLEDO, Columbus, OH, USA) [46]. Then, the specimens were immersed in deionized water (10 mL) and stored at 37 °C. Mass and volume of the specimens at each time point (At each time point (3, 6, 24, 48, 120, 168, 336, 504, and 672 h) were recorded without changing the storage solution. The changes in mass (ΔM, wt%) and volume (ΔV, vol%) of the specimens were obtained using the following equations [47].

(4) $$\Delta M\;=\frac{100\left[M_{t}-{\;M}_{0}\right]}{M_{0}}$$

(5) $$\Delta V\;=\frac{100\left[V_{t}-{\;V}_{0}\right]}{V_{0}}$$

where $M_{t}\;$ and $V_{t}$ are the mass and volume of the specimens recorded at time t, respectively. $M_{0}$ and $V_{0}$ are the mass and volume of the specimens that were measured before placing in water.

### 2.6. Color Stability ($\Delta E_{00}$)

The measurement was conducted following the protocol reported in the previous study [48]. Briefly, disc specimens were prepared and immersed in deionized water at a controlled temperature of 37 °C for 7 days. An intraoral dental spectrophotometer (Easyshade V; VITA Zahnfabrik, Baden-Württemberg, Germany) was employed to determine the CIELab coordinates of the specimens before and after immersion in deionized water.

The spectrophotometer was calibrated using the standard VITA Classical shade guide (A3, VITA Zahnfabrik, Baden-Württemberg, Germany). The measurement was performed in a box with a controlled illuminance of ~1000 lux (LX1330B Light Meter; Dr. Meter Digital Illuminance, StellarNet Inc., Tampa, FL, USA). Then, the tip of the spectrophotometer was positioned at the center of the specimens over the standardized photography neutral 18% gray card (Kodak Gray Cards, Rochester, New York, NY, USA). Then, the color coordinates (CIE L*, a*, b*, C*, and h^o^) of the composites were recorded in triplicate. The L*, a*, and b* represent value, red-green, and yellow-blue axes, respectively. C*, and h^o^ were chroma and hue angle. The color differences or color stability ($\Delta E_{00}$) of the specimens after immersion in water were calculated using the CIEDE2000 (E_00_) formula as follows.

(6) $$E_{00}={\left[\left(\frac{\Delta L^{\prime }}{K_{L}S_{L}}\right)+\left(\frac{\Delta C^{\prime }}{K_{C}S_{C}}\right)+\left(\frac{\Delta H^{\prime }}{K_{H}S_{H}}\right)+R_{T}\left(\frac{\Delta C^{\prime }}{K_{C}S_{C}}\right)\left(\frac{\Delta H^{\prime }}{K_{H}S_{H}}\right)\right]}^{1/2}$$

where $\Delta L^{\prime }$, $\Delta C^{\prime }$, $\Delta H^{\prime }$ represent the differences in lightness, chroma, and hue, respectively. $R_{T}$ represents a rotation function related to the interaction between chroma and hue differences in the blue region. Additionally, $S_{L}$, $S_{C}$, and $S_{H}$ are weighting functions, and $K_{L}$, $K_{C}$, $K_{H}$ are the correction terms for experimental conditions [49].

### 2.7. Statistical Analysis

The results in the current study were reported as mean (SD) or median (min-max). The data were analyzed using Prism 9 for macOS version 9.3.1 (GraphPad Software, San Diego, CA, USA). The normality of data was checked using the Shapiro–Wilk test. A one-way ANOVA followed by Tukey multiple comparisons was used for normally distributed data. For data with non-normal distribution, Kruskal–Wallis and Dunn’s tests were employed. The data were considered statistically significant if *p* < 0.05. Additionally, the power of the test was calculated using G*Power for MacOS version 3.1.9.6 (Heinrich Heine University Düsseldorf, Düsseldorf, Germany) [50] using the data from the previous study [9]. The result suggested that using 5 samples for each test should provide power > 0.95 at alpha = 0.05 for a one-way ANOVA.

The current study also used factorial design analysis [10] to assess the effect of increasing concentrations of MCPM (from 3 to 6 wt%) and Sr/F-BGNPs (from 5 to 10 wt%) on the tested properties. The formulation of experimental composites in the current study contained 2 main variables at 2 levels of factorial design. Hence, the factorial equation is given as follows.

(7) $$\ln P=\langle \mathrm{lnP}\rangle \pm b_{1}\pm b_{2}\;\pm b_{1,2}$$

where, $b_{1}$ and $b_{2}$ represent the effect of raising MCPM (from 3 to 6 wt%) and Sr/F-BGNPs (from 5 to 10 wt%) concentrations on the tested property value (P) of the experimental materials, $b_{1,2}$ represents an interaction effect from the increasing concentration of MCPM and Sr/F-BGNP, the bracket indicated the average value of lnP. Additionally, the percentage effect of variables (Q, %) can be obtained using Equation (8).

(8) $$Q\;=100\;\left(1-\frac{G_{H}}{G_{0}}\right)=100\left(1-\exp\left(2b_{i}\right)\right)$$

where, $G_{H}\;$ and $G_{0}$ represent the geometric average properties for the two formulations containing high-level additives versus the other two formulations with low-level additives, respectively. The *b* values from Equation (6) are denoted by $b_{i}$. The effect of the rising concentration of MCPM was therefore obtained by comparing the geometric average of S10M6 and S5M6 with that of S10M3 and S5M3. Additionally, the effect from Sr/F-BGNPs was determined by the geometric average of S10M6 and S10M3 over S5M6 and S5M3. The interaction effects are then calculated from the geometric average of S10M6 and S5M3 over that of S10M3 and S5M6. The main effects and interaction effects were considered significant if the b values were greater than its 95% confidence interval (95% CI).

## 3. Results

### 3.1. SEM-EDX of Sr/F-BGNPs and MCPM

**Figure 1:**
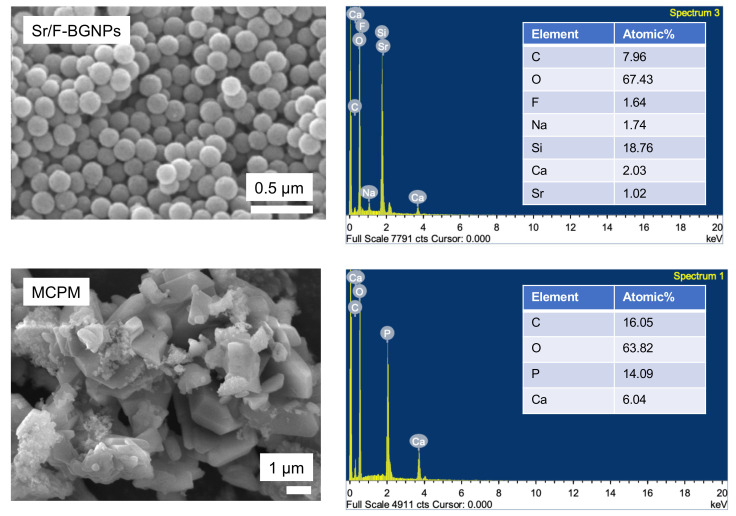
SEM images and EDX results of Sr-BGNPs and MCPM, respectively.

The spherical monodispersed Sr/F-BGNPs were successfully synthesized using the sol-gel process (Figure 1). SEM images suggested that the particle size of Sr/F-BGNPs was ~200 nm. Additionally, F and Sr were detected in the particles. SEM and EDX results of MCPM also demonstrated that the Ca/P atomic ratio of MCPM is ~0.5.

### 3.2. Degree of Monomer Conversion (DC)

**Figure 2:**
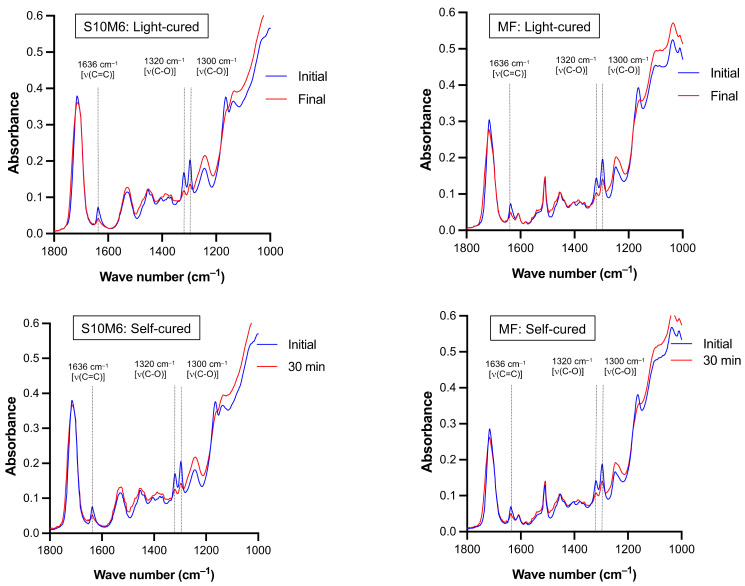
The representative FTIR spectra of experimental core build-up material (S10M6) and MF upon light-curing and chemical-curing. Peaks at 1300 and 1320 cm^–1^ [ν (C-O)] and 1636 cm^–1^ [ν (C=C)] of methacrylate groups [51] were reduced upon polymerization.

**Figure 3:**
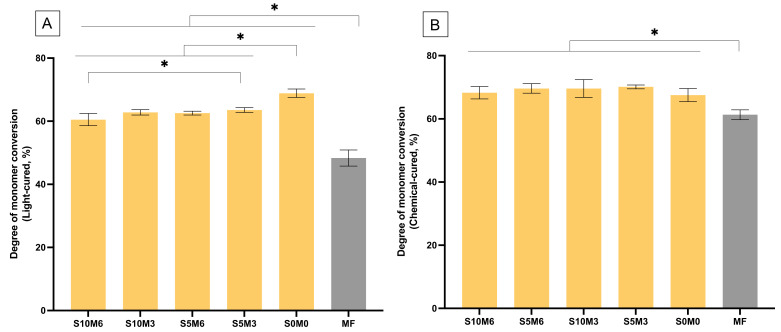
Degree of monomer conversion upon (**A**) light-activation and (**B**) chemical activation. Stars indicate *p* < 0.05. Error bars are SD (*n* = 5).

The reduction of peaks at 1300 and 1320 cm^–1^ [ν (C-O)] and 1636 cm^–1^ [ν (C=C)] of the experimental materials (S10M6) and MF were detected in both light-curing or self-curing mechanisms (Figure 2). For DC upon light-curing, the highest and lowest results were detected with S0M0 (68.9 ± 1.2%) and MF (48.3 ± 2.3%), respectively (Figure 3A). The DC of MF was significantly lower than the experimental materials (*p* < 0.01). The DC of S0M0 was significantly higher than that of S10M6 (65.5 ± 1.0%), S5M6 (62.58 ± 0.8%), S10M3 (62.6 ± 0.5%), and S5M3 (63.5 ± 0.7%) (*p* < 0.01). Additionally, the S5M3 exhibited a significantly higher DC than S10M6 (*p =* 0.0242). Furthermore, the factorial analysis showed that the increase of MCPM and Sr/F-BGNPs concentrations decreased DC upon light-activation by approximately 2.2 ± 1.6% and 2.6 ± 0.9%, respectively.

**Figure 4:**
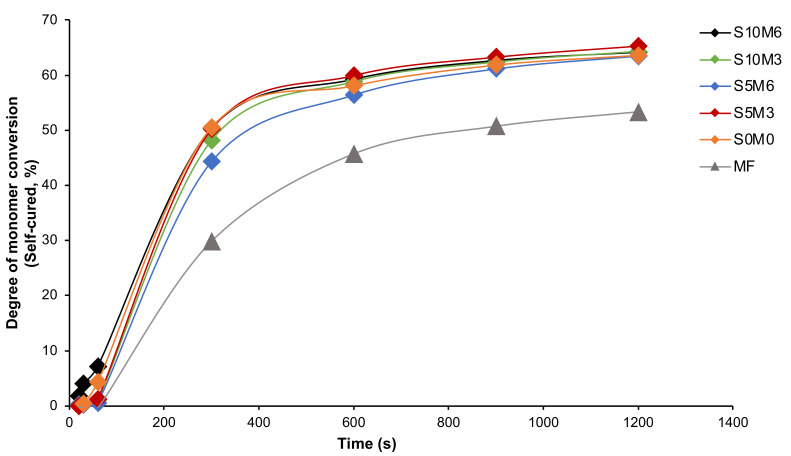
The degree of monomer conversion (self-cured) versus time (20, 30, 60, 300, 600, 900, and 1200 s) from the representative sample.

For chemical-activated DC, the highest and lowest values were detected from S5M3 (70.1 ± 0.5%) and MF (61.3 ± 1.4%), respectively (Figure 3B). The DC of MF was also significantly lower than that of all experimental materials (*p* < 0.05). The DC amongst S10M6 (68.3 ± 1.7%), S5M6 (69.6 ± 1.3%), S10M3 (69.6 ± 2.5%), S5M3, and S0M0 (67.5 ± 1.9%) were comparable (*p* > 0.05). The self-cured DC of the experimental materials increased more rapidly than that of MF (Figure 4). Additionally, the factorial analysis indicated that the increase of MCPM slightly reduced DC upon chemical activation by approximately 1.3 ± 0.9%, whilst the effect from increasing Sr/F-BGNPs concentration was negligible.

### 3.3. Biaxial Flexural Strength (BFS) and Modulus of Elasticity (BFM)

**Figure 5:**
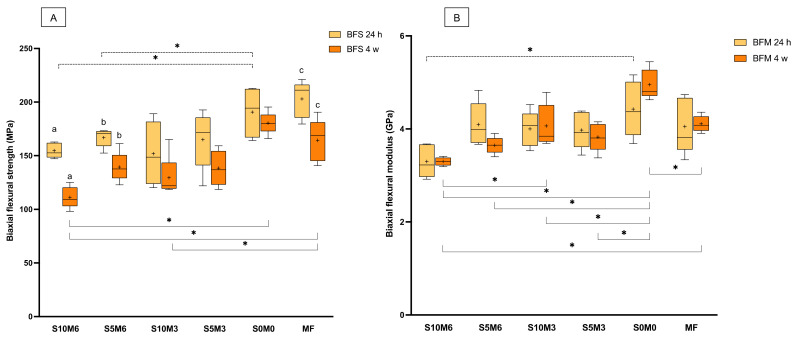
(**A**) Biaxial flexural strength and (**B**) biaxial flexural modulus of materials at 24 h and 4 weeks. The boxes indicate the first quartile (Q1) to the third quartile (Q3), the horizontal line in the boxes represents the median, the whiskers represent the maximum and minimum values, and “+” represents the mean value (*n* = 5). The same letters represent *p* < 0.05 of the data compared within the same materials at 24 and 4 weeks. The dashed and solid lines with stars indicated *p* < 0.05 of data compared among different materials at 24 h and 4 weeks, respectively.

The highest and lowest BFS values at 24 h were obtained from MF (203 ± 15 MPa) and S10M3 (152 ± 26 MPa), respectively (Figure 5A). The BFS of MF was significantly higher than that of S10M3 (*p* = 0.0074) and S10M6 (155 ± 6 MPa) (*p* = 0.0124). The BFS of MF was similar to that of S0M0 (191 ± 20 MPa) (*p* = 0.9301), S5M3 (165 ± 24 MPa) (*p* = 0.0724), and S5M6 (167 ± 8 MPa) (*p* = 0.0971). The BFS of all materials was decreased at 4 weeks. The significant differences between the BFS at 24 h and 4 weeks were detected with S10M6 (*p* < 0.01), S5M6 (*p* = 0.0056), and MF (*p* = 0.0104). At 4 weeks, S10M6 (111 ± 10 MPa) showed a significant lower BFS than S0M0 (181 ± 20 MPa) (*p* = 0.0007) and MF (164 ± 20 MPa) (*p* = 0.0143). Additionally, the BFS of MF was significantly higher than that of S10M3 (130 ± 20 MPa) (*p* = 0.0484) but comparable to that of S0M0 (181 ± 10 MPa) (*p* > 0.05), S5M6 (140 ± 14 MPa) (*p* > 0.05), and S5M3 (140 ± 16 MPa) (*p* > 0.05). The factorial analysis showed no significant effects on BFS at 24 h upon raising the concentrations of MCPM and Sr/F-BGNPs. At 4 weeks, the increase of MCPM and Sr/F-BGNPs levels reduced BFS by 6.3 ± 6.1% and 12.9 ± 9.4%, respectively.

S0M0 (4.4 ± 0.5 GPa) showed the highest BFM, while the lowest value was detected with S10M6 (3.3 ± 0.3 GPa) (Figure 5B). The BFM of MF (4.1 ± 0.5 GPa) was similar to that of S10M6 (*p* = 0.1650), S5M6 (4.1 ± 0.4 GPa) (*p* > 0.99), S10M3 (4.0 ± 0.3 GPa) (*p* > 0.99), S5M3 (4.0 ± 0.4 GPa) (*p* = 0.9998), and S0M0 (*p* = 0.8106). Additionally, the BFN of S10M6 was significantly lower than that of S0M0 (*p* = 0.0115). At 4 weeks, the significant reduction of observed BFM was not detected (*p* > 0.05). The BFM of S0M0 (5.0 ± 0.3 GPa) at 4 weeks was significantly higher than that of S10M6 (3.3 ± 0.1 GPa) (*p* < 0.01), S10M6 (3.6 ± 0.1 GPa) (*p* < 0.01), S10M3 (4.1 ± 0.4 GPa) *(p* = 0.0005), S5M3 (3.8 ± 0.3 GPa) (*p* < 0.01), and MF (4.1 ± 0.2 GPa) (*p* = 0.001). Additionally, S10M6 showed a significantly lower BFM than S10M3 (*p* = 0.0029) and MF (*p* = 0.0016). Factorial analysis showed that the increase in MCPM and Sr/F-BGNPs concentrations reduced BFM at 24 h by 7.4 ± 7.2% and 9.4 ± 7.5%, respectively. At 4 weeks, the increase in MCPM concentration decreased BFM by 11 ± 6.5%, whilst the effect from rising Sr/F-BGNPs concentration was negligible.

**Figure 6:**
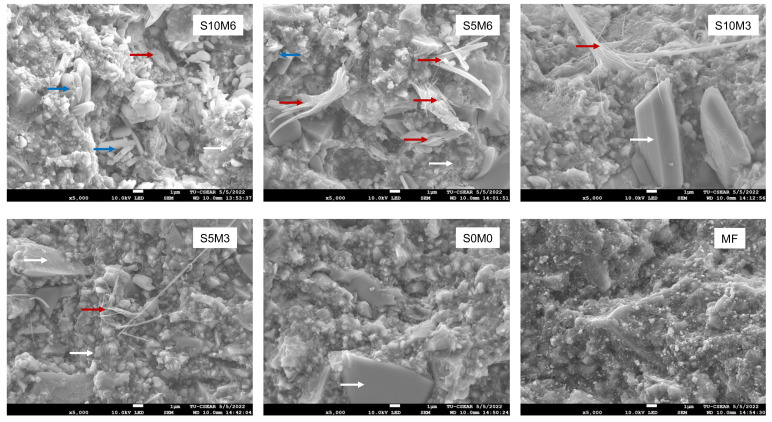
The SEM images of the fracture surface of the representative specimens after the BFS testing. MCPM (blue arrows) and precipitation of calcium phosphates (red arrows) were observed on the experimental core build-up material containing MCPM and Sr-BGNPs. (White arrows) are borosilicate glass [52] and resin matrix.

**Figure 7:**
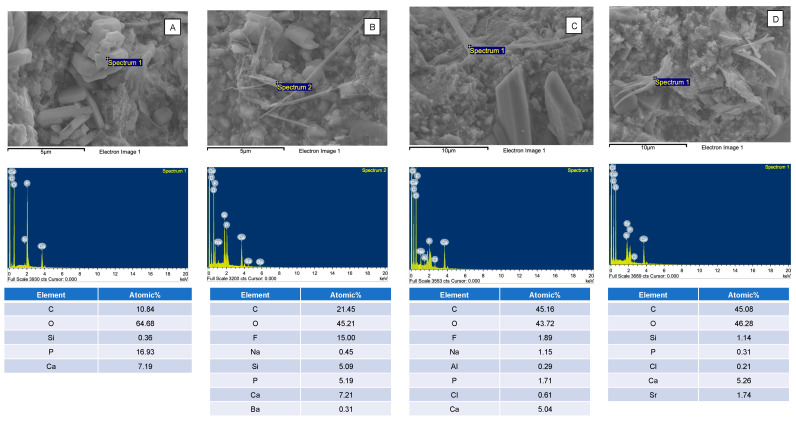
The elemental composition of (**A**) MCPM and (**B**–**D**) calcium phosphates containing F or Sr in the fracture surface of the representative specimens. The calcium phosphate precipitates were only observed in the specimens containing MCPM and Sr/F-BGNPs.

SEM images of the fractured specimens revealed MCPM particles and calcium phosphate precipitation in the glass filler-polymer matrix of the composite (Figure 6). Additionally, EDX results indicated that the precipitates contained various ions such as Sr and F (Figure 7).

### 3.4. Surface Microhardness

**Figure 8:**
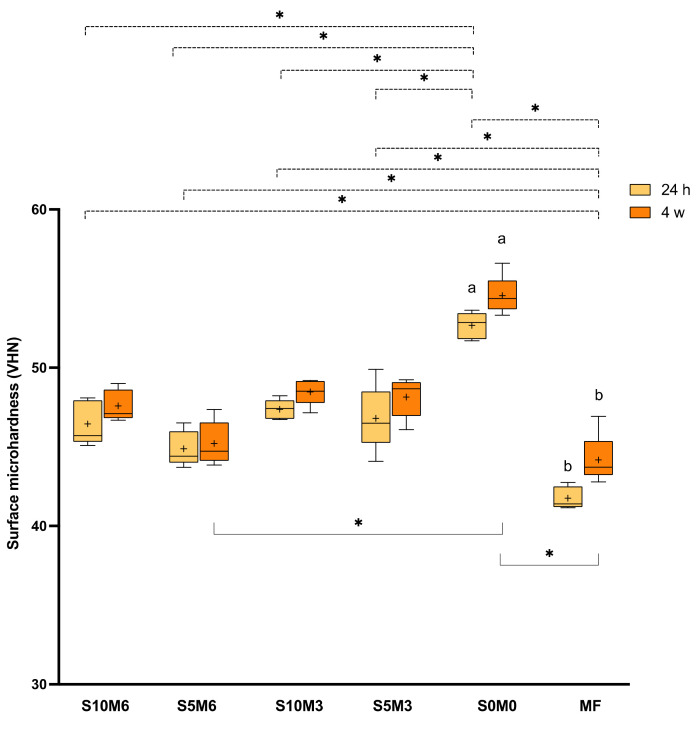
Vickers surface microhardness (VHN) after immersion in deionized water for 24 h. The boxes represent the first quartile (Q1) to the third quartile (Q3), the horizontal line in the boxes represents the median, the whiskers represent the maximum and minimum values, and “+” represents the mean value (*n* = 5). The same letters represent *p* < 0.05 of the data compared within the same materials at 24 h and 4 weeks. The dashed and solid lines with stars indicated *p* < 0.05 of data compared among different materials at 24 h and 4 weeks, respectively.

The highest and lowest Vickers surface microhardness values were obtained from S0M0 (52.7 ± 0.8 VHN) and MF (41.8 ± 0.7 VHN), respectively (Figure 8). MF exhibited a significantly lower surface microhardness than all experimental materials (*p* < 0.01). Additionally, the surface microhardness of S0M0 was significantly higher than that of S10M6 (46.4 ± 1.4 VHN) (*p* < 0.01), S5M6 (44.9 ± 1.1 VHN) (*p* < 0.01), S10M3 (47.4 ± 0.6 VHN) (*p* < 0.01), S5M3 (46.8 ± 2.1 VHN) (*p* < 0.01). Furthermore, the S5M6 showed a significantly lower surface microhardness than S10M3 (*p =* 0.038).

At 4 weeks, a significant increase in surface microhardness was detected with S0M0 (*p =* 0.063) and MF (*p* = 0.0088). The surface microhardness of S0M0 (54.6 ± 1.2 VHN) was significantly higher than that of S5M6 (45.2 ± 1.4 VHN) (*p* = 0.0056) and MF (44.2 ± 1.6 VHN) (*p* = 0.0003). The values of S0M0 were similar to that of S10M6 (47.6 ± 1.0 VHN) (*p* = 0.2663), S10M3 (48.5 ± 0.8 VHN) (*p* > 0.05), and S5M3 (48.2 ± 1.3 VHN) (*p* > 0.05). Factorial analysis indicated that the increase of MCPM and Sr/F-BGNPs showed minimal effect on surface microhardness at 24 h. At 4 weeks, the increase of MCPM reduced the microhardness by approximately 4.0 ± 1.4%, whilst the effect from Sr/F-BGNPs was negligible.

### 3.5. Mass and Volume Changes

**Figure 9:**
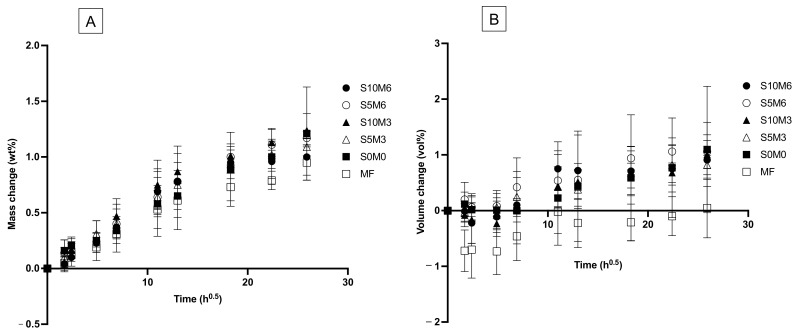
Plots of (**A**) mass change and (**B**) volume changes versus immersion time (square root of an hour) for all materials after immersion in de-ionized water for up to 8 weeks. Error bars are SD (*n* = 5).

**Figure 10:**
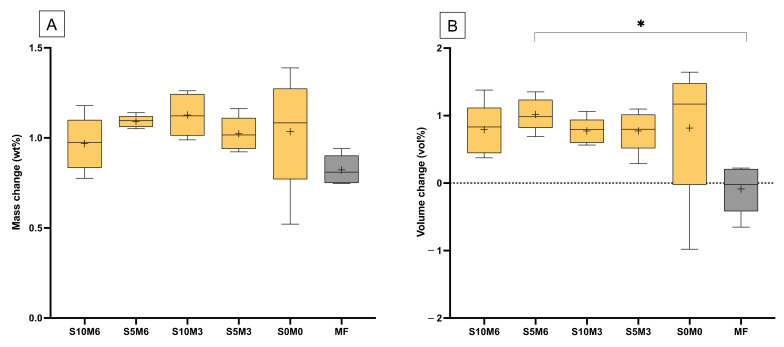
(**A**) The averaged mass change and (**B**) volume change at a late time (336, 504, and 672 h). The boxes represent the first quartile (Q1) to the third quartile (Q3), the horizontal line in the boxes represents the median, the whiskers represent the maximum and minimum values, and “+” represents the mean value (*n* = 5). Star indicated *p* < 0.05.

The mass of all materials was linearly increased upon immersion in water for up to 1 week (Figure 9A). Then, the mass changes began to level off. The mass changes at the late time of all materials are comparable (*p* > 0.05) (Figure 10A). The highest value was obtained from S10M3 (1.13 ± 0.12 wt%) which was not significantly different from that of S5M6 (1.09 ± 0.03 wt%) (*p* = 0.9993), S0M0 (1.03 ± 0.32 wt%) (*p* = 0.9399), S5M3 (1.02 ± 0.09 wt%) (*p* = 0.9059), S10M6 (0.97 ± 0.15 wt%) (*p* = 0.6318), and MF (0.82 ± 0.08 wt%) (*p* = 0.0613). The factorial analysis indicated that the rising MCPM concentration decreased mass change by approximately 4 ± 2%, whilst the effect from rising Sr/F-BGNPs concentration was negligible.

The volume of specimens was slightly changed after immersion in the water early (Figure 9B). At the late time, the lowest volume change was obtained with MF (−0.09 ± 0.36 vol%), which was significantly lower than that of S5M6 (1.02 ± 0.24 vol%) (*p =* 0.0277) (Figure 10B). The volume change of S5M6 was comparable to that of S10M6 (0.79 ± 0.238 vol%) (*p* = 0.9792), S10M3 (0.77 ± 0.19 vol%) (*p* = 0.9710), S5M3 (0.77 ± 0.30 vol%) (*p* = 0.9706), S0M0 (0.82 ± 1.04 vol%) (*p* = 0.9876). The factorial analysis indicated no significant effect from rising MCPM and Sr/F-BGNPs concentrations on volume changes of the materials.

### 3.6. Color Stability

**Figure 11:**
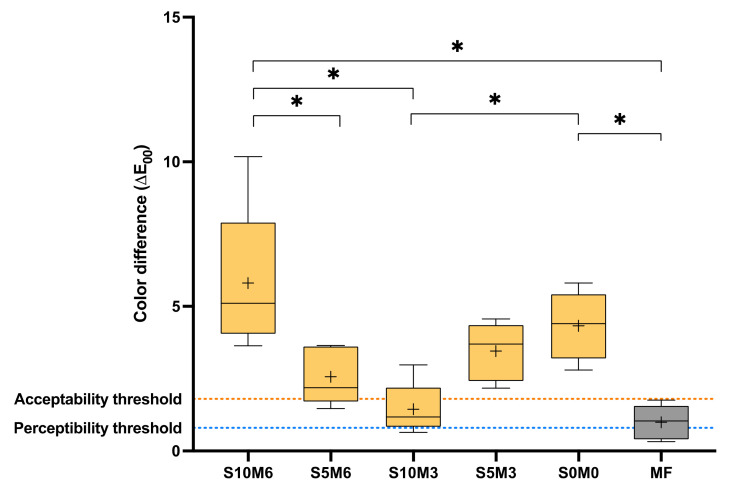
Color difference (∆E_00_) of all material after immersion in water for 1 week. The boxes represent the first quartile (Q1) to the third quartile (Q3), the horizontal line in the boxes represents the median, the whiskers represent the maximum and minimum values, and “+” represents the mean value (*n* = 5). Stars indicated *p* < 0.05.

The highest and lowest observed color difference (∆E_00_) were obtained from S10M6 (5.8 ± 2.6) and MF (1.0 ± 0.6), respectively (Figure 11). The color difference of MF was significantly lower than that of S10M6 (*p* = 0.0281) and S0M0 (4.3 ± 1.2) (*p* = 0.0082). S10M6 also exhibited the significant lower ∆E_00_ than S5M6 (2.6 ± 1.0) (*p* = 0.0108) and S10M3 (1.4 ± 0.9) (*p* = 0.0004). Additionally, S10M3 exhibited a significantly lower ∆E_00_ than S0M0 (*p* = 0.0281). The results from factorial analysis suggested that the increase in MCPM level increased ∆E_00_ by 99 ± 82%, while the effect from rising Sr/F-BGNPs was minimal.

## 4. Discussion

It was reported that bioactive glass nanoparticles and calcium phosphates promoted ion release that could potentially enhance remineralizing actions and prevent dental caries [8,10,14,15]. However, the additives were hydrophilic and non-silanized, which may inevitably reduce the physical/mechanical properties of the composites. The aim of this study was to prepare dual-cured resin composites for core build-up materials added with reactive fillers (MCPM, Sr/F-BGNPs). The effect of MCPM and Sr/F-BGNPs on the tested properties was determined. The results indicated that the addition of MCPM and Sr/F-BGNPs affected light-activated polymerization, biaxial flexural strength, surface microhardness, and color stability of the materials. Hence, the research hypothesis was rejected.

### 4.1. Degree of Monomer Conversion (DC)

In general, composites with high DC may exhibit a low risk of releasing the unreacted toxic monomers from the materials [53]. The concerns of released monomers included cytotoxic, teratogenic, estrogenic, mutagenic, genotoxic effects, and allergic reactions depending upon the released substances [54]. Additionally, it was demonstrated that monomers released from resin composites might interfere with biofilm metabolism, leading to biofilm dysbiosis or an increase in cariogenicity [31]. This may subsequently enhance the risk of caries formation around the composites.

The results showed that the additives slightly reduced the DC of the experimental materials due probably to the mismatch between the refractive index (RI) of fillers and monomers. The RI of fillers was expected to fall within 1.4–1.5 [55] to match with the RI of the monomer mixture such as UDMA/TEGDMA (RI~1.45) [10]. Although the actual refractive indices of MCPM and Sr/F-BGNPs are not known, the result indicated that the effect of the additives on the reduction of DC upon light activation was minimal (~2%) compared to that reported in the previous studies (~4–10%) [8,10]. This could be due to the lower concentration of the additives used in the current study compared to the previous studies. The ability of a material to polymerize upon chemical activation is also essential for core build-up materials to promote adequate polymerization in the deep cavity where light transmission is limited [56]. The results showed that all experimental materials exhibited similar DC upon self-curing regardless of the level of additives. This may be because the chemical-activated polymerization was mainly governed by the type of monomers and level of initiator/activator contained in the materials [57]. The DC upon chemical activation of all materials except for S0M0 was higher than that upon light-curing DC by ~8%. A possible reason could be that the self-curing conversion of the materials was not affected by light penetration.

The experimental materials showed a more rapid increase in DC with time and higher DC values than MF. The actual composition of MF was unknown, so a direct comparison of the different components may not be entirely possible. However, we speculated that the higher DC of experimental materials could be due to UDMA as the primary bulk monomer in the experimental material, while the bulk monomer of MF is bis-GMA. It was reported that UDMA monomers tend to exhibit a higher rate of polymerization than bis-GMA monomers [58,59,60], which may be due to the greater flexible structure of UDMA compared to bis-GMA. The monomer mixture containing low glass transition temperature (*T_g_*) (UDMA, *T_g_* = −35 °C) usually enables higher DC compared with the high *T_g_* monomer (Bis-GMA, *T_g_* = −8 °C) [61]. Additionally, the polymerization of UDMA can be enhanced via the chain transfer reactions of -NH- groups in the molecule [62].

The DC of experimental composites upon light- or chemical-activated polymerization was similar to that of the commercial core build-up materials (50–70%) [3]. The minimum requirement for DC of resin composites in the ISO (International Organization for Standardization) standard is not yet established. However, it is suggested that a conversion greater than 50% for dimethacrylate composites may be sufficient to bind all monomers within the resin matrix [28]. This may help prevent toxic monomer release. The monomer releasing study should be tested in future work.

### 4.2. Biaxial Flexural Strength (BFS) and Modulus (BFM)

Resin composites for core build-up material should provide sufficient mechanical properties to withstand the occlusal loads during functions. The strength of materials was primarily governed by filler characteristics, such as filler load, filler type, size and geometry, and salinization [63]. The previous study employed large particle MCPM (diameter of ~ 50 μm) with high concentration (10–20 wt%) as ion-releasing fillers to enable the desirable mineralizing effect for the composite [10]. However, the main limitation is the significant reduction in the strength of the materials. The addition of reactive fillers in the current study slightly reduced the strength of the materials. This could be due to smaller particles (200 nm and 10 μm) of reactive filler, which may enhance mechanical strength [64]. Additionally, the use of hybrid inorganic fillers consisting of different particle sizes (0.7 μm, 7 μm, and 180 nm) may help maximize filler packing, which could potentially enhance the mechanical strength of the experimental materials [65].

The reactive fillers reduced the strength of the composites. This could be due to the lack of silanization of MCPM and Sr/F-BGNPs, causing poor interaction between the fillers and resin-matrix. This may negatively affect stress transfer between matrix and fillers, thus reducing the strength of the materials [66]. The reactive fillers were not silane treated due to concern regarding the reduction in ion release of the materials. Additionally, the composite paste was prepared by hand mixing. Hence, it is possible that the small fillers, such as Sr/F-BGNPs, may be agglomerated [67]. This may lead to poor dispersion of fillers in the matrix and affect the strength of composites. The fracture surface revealed the MCPM particles in the bulk of materials. It was expected that the MCPM particles should be mostly dissolved after four weeks due to their low Ca/P ratio (~0.5). The detection of multiple MCPM particles in the composite could be due to the polymer network’s high cross-linking, reducing the water diffusion into the materials. The slow dissolution of MCPM may reduce the adverse effects on materials’ physical/mechanical properties, but this may limit the ion release and remineralizing actions of the materials. Additionally, the detection of precipitates containing Sr or F may suggest that the released ions may react and precipitate as plate-like crystals [68,69] inside the bulk of the materials. The formation of new precipitate may help fill voids or defects inside the materials. This may help reduce crack propagation and maintain the mechanical strength of the materials [42].

The flexural strength recorded from the commercial material in the current study was higher than that reported in the previous studies [70,71]. The specimens in the current study were allowed for post-polymerization after light curing for 24 h prior to the immersion for another 24 h. The previous study showed that the DC of dual-cured resin composites activated by light-curing or self-curing modes was increased by ~10% at 24 h [31]. The continuation of polymerization may potentially enhance the cross-linking and mechanical strength of the material [33,62]. The flexural strength of the experimental materials at 24 h or 4 weeks passed the requirement of the standard (>80 MPa) (BS EN ISO 4049:2019 Dentistry-Polymer-based restorative materials) [72]. Although the flexural strength test in the current study was the biaxial flexural strength (BFS) test, the results from the BFS test were expected to be comparable with lower variation than that of the 3-point bending test indicated by the ISO [73]. The main limitation in the current study was that the specimens were not subjected to thermocycling, which could help assess the long-term mechanical performance of the materials.

### 4.3. Surface Microhardness

The high surface microhardness of composites may improve the wear resistance of the materials. The surface microhardness of resin composites usually correlated well with the polymerization and cross-linking of the materials [74]. This was in agreement with the result of the current study. The high surface microhardness value obtained from S0M0 could be due to the higher level of DC upon light-curing or the higher proportion of rigid inorganic glass fillers of the material compared to other experimental formulations. The surface microhardness values obtained from the materials in the current study were similar to that of commercial materials reported in a published study [75]. The increase in surface hardness at four weeks may be due to the post-cure polymerization that could promote the cross-linking of the polymer network [75]. The addition of hydrophilic fillers in the experimental materials may encourage water plasticization of the resin matrix. Hence, the increase of hardness in experimental materials containing MCPM and Sr/F-BGNPs was not significant. The surface microhardness was measured from the top surface where the materials were directly exposed to light-curing. The future work may additionally assess the hardness value at the bottom surface at a greater depth at different time points. This would provide more information regarding the light and self-cure polymerization efficacy of the materials.

### 4.4. Mass and Volume Changes

The mass and volume of the materials are associated with complex behaviors, which are controlled by the equilibrium between component dissolution and water absorption of materials [76]. It was proposed that the changes in mass and volume of composites containing calcium phosphates due to water sorption were linearly increased with the square root of an hour for up to one week, indicating a diffusion control mechanism [28]. This was similar to the results observed in the current study. The level of mass and volume increase (~1 wt% and 1 vol%) of the composite were also lower than that reported in the previously developed composites (~5 wt% and 5 vol%) [8,10]. It was demonstrated that the volume of composites containing 10 wt% of calcium phosphate (MCPM and tricalcium phosphate) was increased by ~3% upon water sorption [28]. Hence, it was expected that the composites containing 3–6 wt% of MCPM should exhibit changes of mass or volume in the range of ~1%. This was in accordance with the result of the current study.

The mass and volume changes observed amongst experimental formulations were comparable. A possible reason could be the use of a low level of MCPM (2 g/100 mL). Another possible reason could be the high-level DC of the materials, which may limit water diffusion into the materials. Hence, the volume of experimental materials was slightly changed. Ideally, the expansion should match the polymerization shrinkage of composites to relieve the shrinkage stress. However, the immediate shrinkage upon polymerization of the composites was known. Future work should employ the bonded-disk method [77] to determine shrinkage immediately after curing the composites and compare it with the volume expansion of the materials.

### 4.5. Color Stability

The color changes of resin-based restorative materials were controlled by various factors such as filler size, aging, water sorption, voids or defects, and level of cross-linking of the polymer network [78,79]. The stability of color of core build-up materials is desirable to ensure predictable esthetic outcomes. It was reported that the color difference (∆E_00_), which is visually identifiable (PT, perceptibility threshold) by the viewer, was greater than 0.8. However, acceptable changes (AT, acceptability threshold) were observed when the color difference (∆E_00_) was less than 1.8 [80]. Hence, the materials should exhibit a color difference (∆E_00_) value within 0.8–1.8.

It was demonstrated that the exposure of large glass fillers on the surface of composites might increase irregularities or roughness on the surface upon aging [52]. This could promote pigment deposition and negatively affect the material’s optical properties and color changes [79,81]. This may be the possible reason for the high ∆E_00_ observed with experimental materials. The results suggested that S10M3 and MF exhibited color changes within the acceptable range. The lower filler content of glass fillers (~55 wt%) in MF [82] compared with the experimental materials may help reduce surface irregularities, which could influence the color and optical properties of the material. For S10M3, the high proportion of spherical-shaped Sr/F-nanoparticles (200 nm) may help reduce surface irregularity and improve optical properties [20], which could reduce the color difference [83]. The use of MCPM at a low level may also reduce water sorption, affecting the color stability of the materials [84].

It should be mentioned that the current study was an in vitro study. Hence, the clinical relevance should be carefully interpreted. In general, the experimental dual-cured resin composites for core build-up materials with the additives (MCPM and Sr/F-BGNPs) at the designated concentrations showed acceptable physical/mechanical properties. Additionally, only S10M3 exhibited satisfactory color stability, which may be considered a suitable candidate formulation for future studies. Further remineralizing or antibacterial tests are needed to optimize the candidate formulations that exhibit anti-caries actions.

## 5. Conclusions

The experimental dual-cured resin composites for core build-up material containing Sr/F-BGNPs and MCPM provided a higher degree of monomer conversion than the commercial material. The additives reduced the biaxial flexural strength, surface microhardness, and color stability of the experimental composite. However, the values remained within the satisfactory range. The increase in the concentration of additives exhibited no detrimental effects on the physical and mechanical properties of the experimental materials. However, the materials’ anti-caries effects need to be examined in future studies.

## Figures and Tables

**Table 1:** Composition of initiator and activator liquids for composites in the current study. The concentration is in wt%.

Liquid Formulations	UDMA	TEGDMA	HEMA	10-MDP	BP	CQ	DMPT
Initiator liquid	70	23.25	3	3	0.75	0.5	0
Activator liquid	70	22.25	3	3	0	0.5	0.75

**Table 2:** Formulations of experimental materials. The concentration is in wt%.

Formulations (Particle Diameter)	Borosilicate Glass (7 μm)	Borosilicate Glass (0.7 μm)	Borosilicate Glass (180 nm)	Sr/F-BGNPs (200 nm)	MCPM (10 μm)
S10M6	50.4	25.2	8.4	10	6
S5M6	53.4	26.7	8.9	5	6
S10M3	52.2	26.1	8.7	10	3
S5M3	55.2	27.6	9.2	5	3
S0M0	60	30	10	0	0

**Table 3:** Composition of the commercial material. The actual composition was not provided by the manufacturer.

Name	Composition	Lot No.	Supplier
MultiCore Flow (MF)	Base: 10–20 wt% Ytterbium trifluoride, 2.5–20 wt% bisphenol A-glycidyl methacrylate (Bis-GMA), 2.5–10 wt% Urethande dimethacrylate (UDMA), 2.5–10 wt% Triethylene glycol diethacrylate (TEGDMA)	20074X	Ivoclar Vivadent AG, Schaan, Liechtenstein
	Catalyst: 10–20 wt% Ytterbium trifluoride, 2.5–20 wt% bisphenol A-glycidyl methacrylate (Bis-GMA), 2.5–10 wt% Urethande dimethacrylate (UDMA), 2.5–10 wt% Triethylene glycol diethacrylate (TEGDMA), 0.1–1 wt% dibenzoyl peroxide		

## Data Availability

The datasets generated and/or analyzed during the current study are available from the corresponding author on reasonable request.
